# Tear Film Changes and Ocular Symptoms Associated with Soft Contact Lens Wear

**DOI:** 10.3390/vision9020027

**Published:** 2025-04-01

**Authors:** Eduardo Insua Pereira, Madalena Lira, Ana Paula Sampaio

**Affiliations:** 1 Physics Center of Minho and Porto Universities (CF-UM-UP), School of Sciences, University of Minho, 4710-057 Braga, Portugal; 2 Centre of Molecular and Environmental Biology (CBMA), University of Minho, 4710-057 Braga, Portugal

**Keywords:** tear film, contact lenses, ocular symptoms, non-invasive break-up time

## Abstract

Discomfort is one of the leading causes associated with contact lens dropout. This study investigated changes in the tear film parameters induced by lens wear and their relationship with ocular symptomology. Thirty-four lens wearers (32.9 ± 9.1 years, 7 men) and thirty-three non-lens wearers (29.4 ± 6.8 years, 12 men) participated in this clinical setting. Subjects were categorised into asymptomatic (n = 11), moderate (n = 15), or severe symptomatic (n = 8). Clinical evaluations were performed in the morning, including blink frequency and completeness, pre-corneal (NIBUT) and pre-lens non-invasive break-up (PL-NIBUT), lipid interference patterns, and tear meniscus height. Contact lens wearers had a higher percentage of incomplete blinks (37% vs. 19%, *p* < 0.001) and reduced tear meniscus height compared to controls (0.24 ± 0.08 vs. 0.28 ± 0.10 mm, *p* = 0.014). PL-NIBUT was shorter than NIBUT (7.6 ± 6.2 vs. 10.7 ± 9.3 s. *p* = 0.002). Significant statistical differences between the groups were found in the PL-NIBUT (*p* = 0.01) and NIBUT (*p* = 0.05), with asymptomatic recording higher times than symptomatic. Long-term use of silicone–hydrogel lenses can affect tear stability, production, and adequate distribution through blinking. Ocular symptomology correlates with tear stability parameters in both lens wearers and non-wearers.

## 1. Introduction

Hydrophilic contact lenses (CLs) underwent significant development in 1956 with the discovery of hydrogels [1]. In the late 1990s, the introduction of siloxane molecules into the polymer matrix revolutionised CL technology, enabling high oxygen transmissibility [2,3]. However, the first generation of silicone–hydrogel CLs exhibited greater rigidity, reduced wettability, and increased susceptibility to lipid deposition compared to conventional hydrogels [4]. To improve mechanical properties and comfort, advancements such as plasma surface treatments [5] and the incorporation of hydrophilic compounds like poly(vinyl alcohol) (PVA) and poly(vinylpyrrolidone) (PVP) were introduced, enhancing lubricity and wearability [6]. Despite these innovations, biomimetic CLs [2] continue to induce significant biochemical and biophysical alterations in the pre-ocular tear film (POTF) [7]. Lens wear reduces the outer lipid layer of the pre-lens tear film compared to the naïve POTF [8,9], increasing tear evaporation [10] and facilitating lipid interactions with the CL surface [11]. This can lead to the formation of non-wettable areas, compromising tear dispersion and stability [12,13]. Additionally, the presence of a lens over the cornea alters neural sensitivity [14], affecting tear production and its adequate distribution through blinking [14,15]. Reduced tear volume and disrupted tear film composition lead to increased shear forces during blinking, potentially causing conjunctival lid epitheliopathy, corneal epithelial damage, nociceptor stimulation, and discomfort [16,17,18,19]. If not addressed, this inflammatory cycle can result in ocular discomfort and the development of dry eye disease (DED) [16,17,18,20], with CL wearers facing a two- to three-fold increased risk compared to non-wearers [21]. Ocular discomfort related to dryness is a primary reason for CL discontinuation, with approximately 50% of wearers experiencing symptoms or signs of DED [22,23]. Despite its prevalence, the precise clinical features underlying CL-induced discomfort (CLD) remain unclear, as the complex interactions between lens materials and the ocular surface are not yet fully understood. The Tear Film and Ocular Surface Society (TFOS) [24] has highlighted the need for further clinical trials to comprehensively investigate this issue and guide the development of new materials and strategies to mitigate CLD. While numerous studies have explored the impact of CL wear on ocular surface health and discomfort [7,11,18,19,25,26,27,28,29,30,31,32,33,34,35], limited scientific evidence differentiates levels of ocular discomfort and correlates these findings with novel CL materials [25,26,29,33]. As such, this study aims to provide a comprehensive analysis of POTF and blinking changes associated with prolonged biomimetic CL wear, considering distinct ocular symptomatology profiles. Additionally, this research highlights the potential role of CL technological advancements—such as polymer formulations and coating—in improving long-term comfort. Understanding these interactions will contribute to the refinement of CL designs and the development of personalised interventions to enhance ocular health and wearer satisfaction.

## 2. Materials and Methods

### 2.1. Study Characterisation

Sixty-seven individuals participated in this case–control clinical trial: thirty-four (34) CL wearers and thirty-three (33) non-wearers. The subjects were screened for refractive and ocular health examinations during their first visit. Exclusion criteria included active eye infection, inflammation or allergy, non-transparent optical media, use of systemic or ocular medication within 12 weeks before and during the study, and history of ocular surgery. Participants were between 18 and 40 years old, with correctable visual acuity of at least 08/10 or better in each eye. Clinical assessments were made during a second visit in the morning (between 10–12 am). Several polymers were identified among the CL wearers, as described in Table 1.

The University of Minho’s Ethics Subcommittee for Life and Health Sciences reviewed and approved the study protocol. This research followed the principles stated in the Declaration of Helsinki [36]. After a thorough explanation of the study procedures, informed consent was obtained from all participants.

### 2.2. Subjects Allocation into Groups

CL wearers and non-wearers were classified into three groups according to their ocular symptomatology: asymptomatic, moderate, and severe symptomatic. The control group consisted of asymptomatic non-lens wearers. This classification was determined by completing the Ocular Surface Disease Index (OSDI) questionnaire. The form comprises 12 questions that rapidly assess ocular symptoms related to DED and its impact on visual function [37]. The total score was presented on a scale from 0 to 100, with higher scores corresponding to greater symptom severity. The index has demonstrated good sensitivity and specificity [38]. The cut-off values of 13 and 36 were considered to distinguish asymptomatic from symptomatic patients and discriminate between moderate and severe symptomology, respectively [38].

### 2.3. Clinical Evaluation and Procedures

The clinical evaluation comprised blinking and tear film characterisation. Blinking was assessed in frequency and completeness, and the tear film was assessed for its structure, stability, and volume. All clinical data were captured using a smartphone digital camera (Xiaomi Inc., Beijing, China) adapted to a slit lamp. After the experimental period, the raw files were downloaded and examined on an iMac (Apple Inc., Cupertino, CA, USA) computer with a 21.5-inch screen. The videos were edited and analysed with iMovie (Apple Inc., Cupertino, CA, USA), and measurements were performed throughout ImageJ 3 Version 8 (U.S. National Institutes of Health, Bethesda, MD, USA). This image processing software allows for accurate calculations of digital photographs, with a margin of error of 0.001 mm [39]. Only the right eye of each participant was considered for the analysis.

#### 2.3.1. Blinking Characterisation

Each participant was instructed to stare at a fixation point 1.5 m away for approximately 2 min. Although candidates knew they were being filmed, they were unaware of the video’s purpose. Initial blinks were discarded from the analysis until a more regular blinking pattern was observed. A blink was considered complete if the upper eyelid entirely covered the ocular surface and touched the lower lid margin [8]. Blink frequency was defined as the total number of blinks per minute, while completeness was expressed as the percentage of incomplete blinks.

#### 2.3.2. Tear Film Characterisation

**Figure 1:**
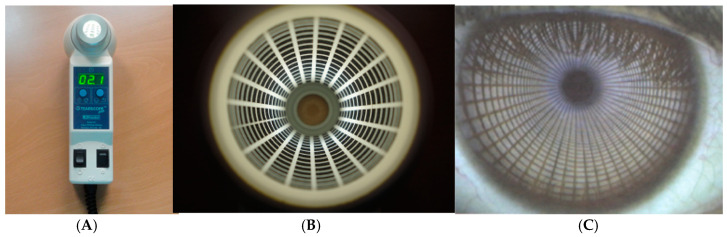
The Tearscope Plus (**A**), the coarse used in the measures (**B**), and the reflection pattern in the subject’s eye (**C**).

Tear film structure, stability, and volume were evaluated with a tearscope (Keeler Instruments Ltd., Windsor, UK—Figure 1A) [40] coupled to the slit lamp using a 16× magnification. Detailed procedures for each parameter are described in subsequent sections.

##### Tear Film Stability

Tear stability was evaluated indirectly by measuring the non-invasive tear break-up time. A mesh filter was projected onto the participant’s pre-corneal surface using the method proposed by Guillon [40]—Figure 1B. Tear break-up time was considered the time in seconds from the last blink to the appearance of the first distortion in the reflected filter—Figure 1C. Each measurement was repeated three times per eye to increase reliability and was made on the subjects’ corneal surface (NIBUT)] and with the lens in situ for the CL wearers (PL-NIBUT)]. The room temperature and humidity were controlled during all examinations.

##### Lipid Layer Characterisation

The lipid interference patterns were used to evaluate the tear film lipid layer. The diffuse light source of the tearscope, viewed under a non-illuminated slit lamp, enabled the observation of the superficial tear lipid layer by interference fringes. The visible patterns were classified into five categories: meshwork, wave, amorphous, coloured, and abnormally coloured fringes [41]. Table 2 describes each category and the corresponding estimated thickness. The classification was performed after video analysis, considering the interval between blinks, the lipid mixture, and the possible level of contamination in the tear.

##### Tear Film Volume

**Figure 2:**
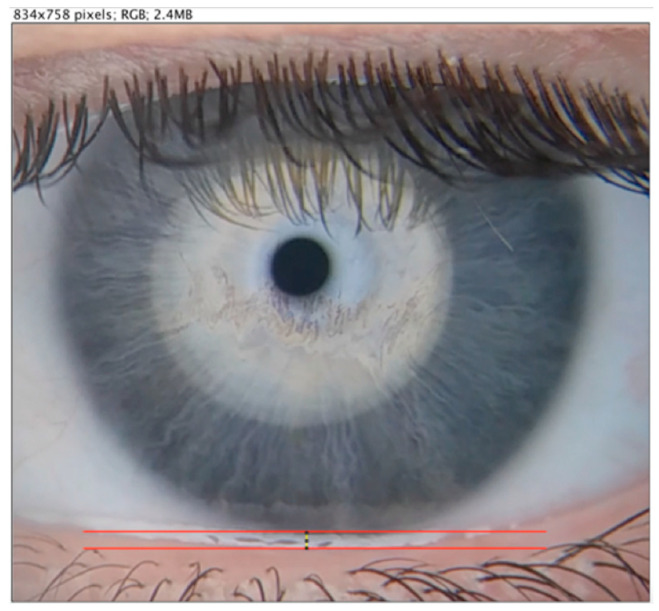
Detail of the digital tear meniscus height measurement using the ImageJ 3 caliper tool.

The inferior prism meniscus represents a reasonable estimation of tear volume, as more than 70% of tears are contained in the structure [41]. Photographs were taken of each participant’s inferior ocular lid margin. The meniscus height was measured as the distance, in millimetres, from the prism reflection line to the edge of the eyelid [25]. Measurements were made using ImageJ and adjusted for magnification (each pixel corresponded to 0.02 mm)—Figure 2.

### 2.4. Statistical Analysis

The statistical analysis was performed using Statistical Package for Social Sciences version 23 (SPSS Inc., Chicago, IL, USA). The *Kolmogorov–Smirnova* test was used to evaluate the normality of the data distribution. The percentage of incomplete blinks (*p* < 0.001), blink frequency (*p* = 0.014), NIBUT (*p* < 0.001), PL-NIBUT (*p* < 0.001) and tear prism meniscus height (*p* = 0.010) were not normally distributed, and comparison among groups was achieved using *Mann–Whitney* U or *ANOVA* tests.

The tear lipid patterns were considered categorical data; the corresponding *chi-squared* test assessed the relationship between variables and the groups. Correlations were established using the *Crammer V* coefficient. The significance level was set at 0.05 (a = 0.05).

## 3. Results

### 3.1. Sample Characterisation

The CL-wearers’ group included 7 men and 27 women (n = 34), with a mean age of 32.9 ± 9.1 years and an average refraction of −3.8 ± 2.2 Dioptres (D). Among them, 11 were considered asymptomatic (OSDI 5.2 ± 4.9), 15 were mildly symptomatic (OSDI 22.0 ± 6.4), and 8 were severely symptomatic (OSDI 40.8 ± 4.3).

The non-lens wearing group included 33 subjects: 11 controls (OSDI 6.2 ± 3.1), 9 mild symptomatic (OSDI 22.5 ± 7.1), and 13 severe symptomatic (OSDI 53.6 ± 9.5). Of these, 12 were male and 21 were female, with a mean age of 29.4 ± 6.8 years and a mean myopia of −1.1 ± 2.0 D. The CL wearers were more myopic than non-wearers (*p* < 0.001, *independent t-test*) and had been wearing lenses for an average of 9.7 ± 7.6 years. Of the CL wearers, 23 used monthly replacement CLs, while the others used daily disposable lenses.

### 3.2. Blinking Characterisation

**Figure 3:**
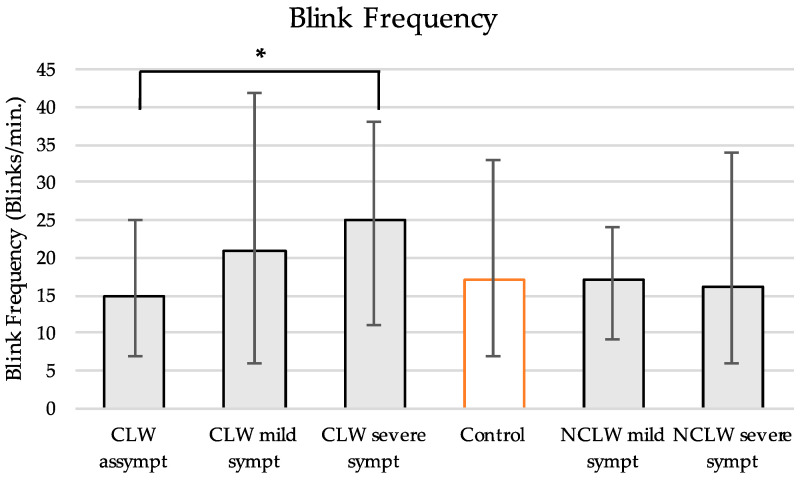
Blink frequency and standard deviation for the contact lens wearers (CLW), non-wearers (NCLW), and controls. * *p* = 0.04.

A total of 1325 and 968 images were analysed for the CL wearers and non-wearers, respectively. Figure 3 represents the mean blink frequency for the subgroups.

Blink frequency increased with symptom severity in the CL wearers (15 ± 6 vs. 25 ± 8, blinks/min., *p* = 0.04, *ANOVA*)—Figure 3. In contrast, the mean frequency remained similar for the non-lens users and controls. Overall, the CL wearers blinked more frequently than the controls (20 ± 9 vs. 17 ± 7 blinks/min.) although the difference was nearly statistically significant (*p* = 0.061, *Mann–Whitney U*). In terms of blink completeness, 488 (37%) and 183 (19%) of the blinks were considered incomplete for the CL wearers and controls, respectively (*p* < 0.001, *Mann-Whitney U*).

### 3.3. Tear Film Stability

The tear stability results for the CL wearers and controls are resumed in Table 3.

The mean PL-NIBUT was lower than the NIBUT (*p* = 0.002, *Mann-Whitney U*)—Table 3.

**Figure 4:**
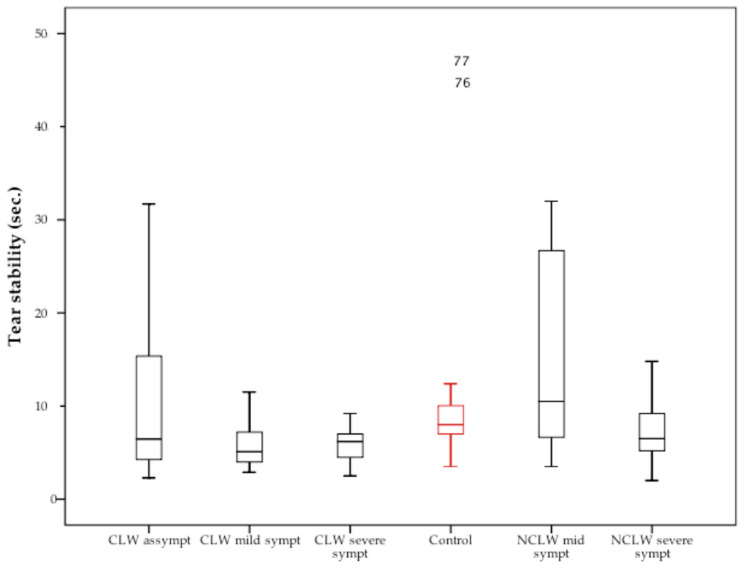
Box-plot representing the non-invasive break-up times for the asymptomatic, mildly, and severely symptomatic contact lens wearers (CLW), non-lens wearers (NCLW), and controls.

Figure 4 describes the data distribution for the CL wearers, non-lens wearers, and controls.

The asymptomatic CL wearers [2.3; 31.7 s] and controls [3.5; 45.9 s] exhibited the broader dispersion data—Figure 4. The CL subgroup analysis revealed a significant difference between the groups (*p* = 0.01, *ANOVA*), with the asymptomatic wearers presenting higher PL-NIBUTs compared to the moderately (11.3 ± 9.7 vs. 5.9 ± 2.5, *p* = 0.004, *ANOVA*) and severely symptomatic groups (11.3 ± 9.7 vs. 5.8 ± 2.0, *p* = 0.025, *ANOVA*). Comparison between the non-lens wearers identified a similar trend (*p* = 0.05, *ANOVA*), with a robust variation observed specifically between the mildly and the severely symptomatic groups (15.0 ± 10.8 vs. 7.4 ± 3.3 s, *p* = 0.01, *ANOVA*).

### 3.4. Lipid Layer Characterisation

**Figure 5:**
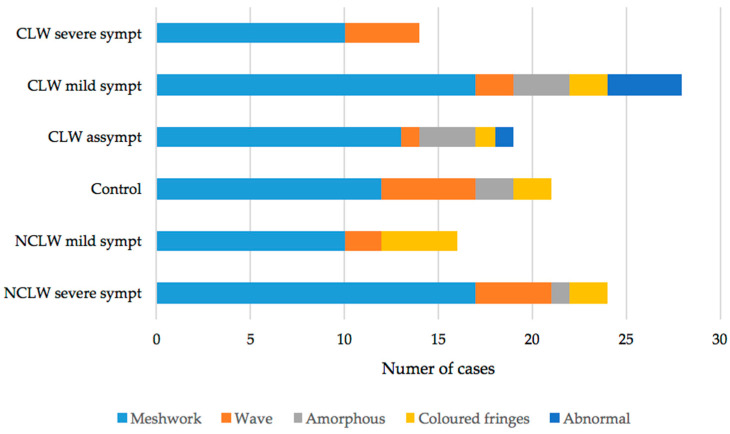
Lipid interference pattern distribution for the asymptomatic, mildly, and severely symptomatic contact lens wearers (CLW), non-lens wearers (NCLW) and controls.

The lipid layer distribution is expressed in Figure 5.

The most frequently observed pattern was the meshwork, with 40 cases among the CL wearers and 39 cases for the non-lens wearers. The wave pattern was identified twice for the non-lens wearers (14) compared to eyes fitted with CL (7)—Figure 5. The distribution was similar across groups (*p* = 0.286, *chi-squared*), and no significant correlations were found between LIP patterns and OSDI scores for either the tested (*p* = 0.499, *Cramer V*) or NCL groups (*p* = 0.452, *Cramer V*).

### 3.5. Tear Film Volume

**Figure 6:**
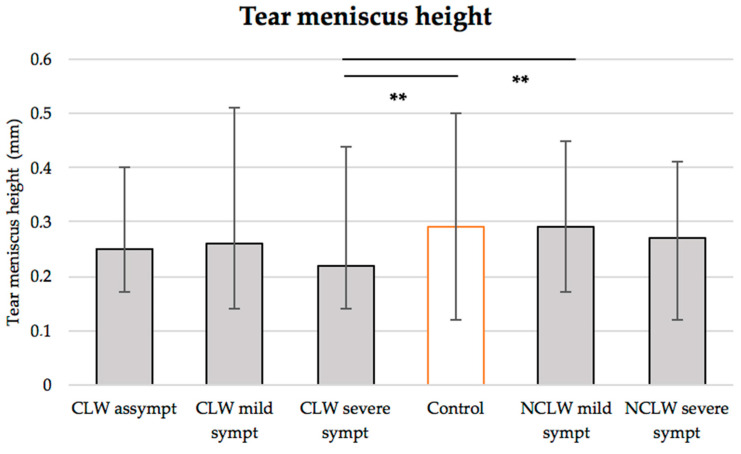
Graphical representation of the tear prism meniscus height for the asymptomatic, mildly, and severely symptomatic contact lens wearers (CLW), non-lens wearers (NLW) and controls. ** 0.01 < *p* < 0.03.

According to the symptomology, Figure 6 shows both groups’ inferior tear prism meniscus height.

The mean tear meniscus height was lower in the CL wearers compared to the controls (0.24 ± 0.08 vs. 0.29 ± 0.14 mm, *p* = 0.014, *ANOVA*). The subgroup analysis also revealed that severely symptomatic CL wearers had lower meniscus height than the controls (0.22 ± 0.09 vs. 0.29 ± 0.14 mm, *p* = 0.016, *ANOVA*) and mildly symptomatic non-CL users (0.22 ± 0.09 vs. 0.29 ± 0.07 mm, *p* = 0.026, *ANOVA*)—Figure 6.

## 4. Discussion

This case–control study proposed an innovative method of analysing tear and blink parameters based on the ocular symptomology profiles of experienced CL wearers and non-wearers. Participants were categorised into groups based on their OSDI scores using the established cut-off points of 13 and 36 to differentiate asymptomatic patients from mild and severe symptoms. These thresholds were initially defined by Schiffman et al. [38], who demonstrated the reliability and validity of the questionnaire in a study of 139 subjects (109 DED patients and 30 controls). Both groups comprised more women than men (79% CL wearers and 64% NCL wearers), allowing a sex-matched comparison, which is relevant given that the population of CL users is primarily female [42]. The CL wearers were significantly more myopic than non-lens wearers (*p* < 0.001), reflecting the frequent use of lenses for ametropia compensation. 

Blinking plays a vital role in maintaining the integrity of the anterior ocular surface. The average blink frequency in humans ranges from 11 to 26 blinks/min [15,43,44,45,46]. within the limits found for the control group in this study—17 ± 7 blinks/min—Figure 3. However, blink characteristics are challenging to quantify, and significant disparities are found between studies depending on the experimental conditions for measurement. CL wear induces changes in the ocular surface, which could elicit ocular discomfort, the primary stimuli for reflex blinking [15]. Previous research has consistently reported increased blink frequencies in both CL wearers [47,48,49] and DED patients [16,50,51]. In this study, lens wearers blinked more frequently than controls, although the difference was marginally non-significant (*p* = 0.061). The relationship between symptoms and CL wear was also demonstrated, with blink frequency being higher in severely symptomatic users compared to asymptomatic users (*p* = 0.04)—Figure 3. The difference may reflect an adaptive ocular response to restore tear instability and prevent evaporation [48]. The percentage of incomplete blinks was substantially more prevalent in CL wearers (*p* < 0.001). While no definitive evidence suggests distinct blinking patterns between lens and non-lens wearers, incomplete blinking seems predominant in symptomatic individuals and is associated with corneal staining [17,47,49]. This underscores the importance of evaluating blink completeness in clinical assessments of ocular discomfort, as its abnormal patterns could serve as an early indicator of ocular surface dysfunction.

The tear NIBUT is influenced by the subject’s anatomical characteristics and environmental conditions, such as temperature and humidity. Differences in NIBUT may also arise from the method used in the analysis, with lower values typically reported by automatic equipment compared to manual methods [52]. Previous clinical studies using the tearscope recorded values ranging from 2.7 to 57.3 s in volunteers aged between 20 and 29 years old [15,41,53,54,55,56], consistent with the control group in this study—10.7 ± 9.3 s—Table 3. Tear stability is related to the structure and thickness of the POTF lipid layer, with lower NIBUTs usually found in individuals with thinner layers [53,57,58,59]. Reduced break-up times are linked with ocular symptoms and DED since an unstable and irregular film increases susceptibility to epithelial desiccation stress [46,51,60]. Differences in the mean NIBUT were observed between groups (*p* = 0.05), with moderately symptomatic individuals displaying higher values than severely symptomatic individuals (*p* = 0.01)—Figure 4. The result suggests that tear film instability correlates with symptom severity, reinforcing its role in diagnosing and managing DED. The analysis of PL-NIBUT values showed a significant reduction in tear break-up times over the CL surface compared to the pre-corneal values (*p* = 0.002)—Table 3. This reduction indicates that the CL insertion results in a thinner pre-lens tear film compared to the POTF, resulting in a premature tear rupture [13,26,61]. Furthermore, the reduced aqueous layer of the tear film enables lipids to interact with the lens, creating non-wetting areas on its surface [13]. Several studies have established an association between reduced tear break-up times and ocular symptoms in CL wearers [13,27,28]. Consistent with these findings, it was shown that asymptomatic wearers reported higher mean PL-NIBUT values compared to moderately (*p* = 0.004) and severely (*p* = 0.025) symptomatic lens wearers—Figure 4. These changes suggest that **PL-NIBUT** measurement provides crucial insights into the interaction between the lens surface and tear film stability, an important factor in ocular discomfort associated with CL wear. Studies investigating these changes, such as those focusing on enhanced lens materials or tailored tear supplementation, could help mitigate ocular discomfort and symptom progression, offering a better experience for contact lens users.

The integrity of the POFT lipid layer depends on inherent factors such as the functionality of the meibomian glands [62], meibum composition [62], blinking effectiveness [15,63], and interpalpebral fissure width [63]. The interference patterns observed with the tearscope allow for a non-invasive observation and analysis of the tear lipid layer with good reproducibility [40,57]. The uniformity and thickness of the layer were estimated using the scale proposed by Guillon [40] who also identified the meshwork pattern as the most prevalent in a sample of 239 individuals with a mean age of 30.2 ± 6.8 years, as opposed to the coloured fringes found in 12% of the sample. Even though the lens presence alters the POTF lipid layer’s characteristics [11,13,29,63], no significant differences were found in the lipid patterns distribution between lens wearers and non-wearers—Figure 5. The meshwork predominant pattern suggests a generalised thinning of the lipids across CL users, and these subtle changes may be complex to quantify by direct slit lamp observation. Advanced computerised techniques for automated tear lipid structure assessment have been proposed to reduce observation time and inter-examiner bias, enhance accuracy, and reduce inter-examiner variability [64,65,66].

According to previous research, the inferior tear meniscus height can vary between 0.23 and 0.32 mm in young adults aged between 21 and 29 years [9,33,41,60,63,67,68]. This parameter can be assessed through slit lamp observation with a reticule eyepiece [15,41], corneal topography [63], or optical coherence tomography [9,33,67,68]. In this work, the menisci were measured through digital photographs and analysed with the ImageJ caliper tool Version 8, a method validated by other researchers [33,68]. The control’s average tear meniscus was within the reported range (0.29 ± 0.14 mm)—Figure 6, with a significantly lower value found for the CL wearers (0.24 ± 0.08, *p* = 0.014). Several investigators also reported this discrepancy, possibly explained by CL-induced hypaesthesia [9,67]. Tear volume is known to decrease throughout the day [30] and can be associated with ocular symptomology [30,31]. As observed in this study, the meniscus height appears to be reduced in symptomatic lens wearers compared to asymptomatic individuals and non-wearers [30,32,69]. However, a significant difference was found only among severely symptomatic wearers, controls (*p* = 0.016) and moderately symptomatic non-wearers (*p* = 0.026)—Figure 6. Chen et al. [30] showed a positive correlation between comfort levels and meniscus height in both symptomatic (n = 20) and asymptomatic (n = 20) lens wearers as well as non-wearers (n = 20) (*p* < 0.05). Similar results were reported by Glasson and colleagues [32], who found that CL habitual users experiencing discomfort had a significantly lower meniscus height than tolerant users (*p* < 0.05). Along with NIBUT and reported symptoms, meniscus volume has been identified as a predictive factor for successful CL fitting.

The following limitations were acknowledged: a small sample size with different characteristics, such as previous CL experience and the type of lenses worn; a single-point assessment, which limited the ability to interpret outcomes prospectively; the use of subjective analysis methods which could introduce observer bias; and the lack of CL wear standardisation, such as maintenance solutions, which may have influenced the results due to the varying interactions between the tear film and different CL materials.

## 5. Conclusions

This study provides a novel perspective by stratifying participants based on ocular symptomatology, allowing for a more precise characterisation of tear film stability and blinking patterns. Additionally, the use of validated digital analysis enhances measurement accuracy, and the detailed evaluation of blinking behaviour provides direct links to ocular discomfort. Prolonged silicone–hydrogel lens wear may impair lacrimal function, destabilize the tear film, and alter blinking patterns. The introduction of biomimetic CL may also influence tissue sensitivity, tear production, and overall tear volume. These findings reinforce the need for individualised CL fitting to improve long-term tolerance.

Beyond clinical implications, future research should explore how environmental factors—such as screen time, humidity, and air quality—impact tear film stability in CL wearers. Advancements in smart CL with biosensors could enable real-time monitoring of ocular health, while nanotechnology-based coatings may enhance tear film compatibility. Interdisciplinary collaborations between optometrists, material scientists, and neuroscientists could drive the development of bioadaptive lenses that dynamically respond to tear film fluctuations. Additionally, investigating the ocular surface microbiome and its interaction with CL may provide insights into discomfort and infection risks.

More extensive longitudinal studies and advanced diagnostic tools are essential to validate these findings and guide the development of tailored interventions, such as improved lens designs or adjunctive therapies. Such innovations promise to enhance long-term comfort and ocular health in lens wearers.

## Figures and Tables

**Table 1:** The main characteristics of the contact lenses worn by the participants.

USAN	Company	FDA Group	EWC (%)	Main Monomers	DK/t (-3D) (Barrer)	Wearing Schedules
**Comfilcon A**	Cooper Vision (San Ramon, CA, USA)	V	48	M3U, FMM, TAIC, IBM, HOB, NMNVA, NVP	160	Monthly
**Lotrafilcon B**	Alcon Vision Care (Fort Worth, TX, USA)	V	33	DMA, TRIS Si Mn	138	Monthly
**Samfilcon A**	Bausch & Lomb (Rochester, NY, USA)	V	46	Si Mn NVP	163	Monthly
**Ocufilcon D**	Cooper Vision	IV	55	pHEMA MA EGDMA	27	Monthly
**Verofilcon A**	Alcon Vision Care	V	51–80	MPDMS GPDMS NVP	100	Daily
**Delefilcon A**	Alcon Vision Care	V	33–80	Si Mn DPMC	156	Daily
**Somofilcon A**	Cooper Vision	V	56	Si Mn TEGDMA	86	Daily
**Etafilcon A**	Johnson & Johnson (New Brunswick, NJ, USA)	IV	58	pHEMA MA	25.5	Daily
**Nelfilcon A**	Alcon Vision Care	II	69	PVA; HPMC; PEG	26	Daily

**USAN**: United States adopted name; **FDA**: Food and Drugs Administration; **EWC**: equilibrium water content; **DK/t**: oxygen transmissibility (units: X 10—9 (cm/s) (mL O_2_/mL.mm Hg)); **DMA**: N,N-Dimethylacrylamide; **DMPC**: phosphatidylcholine; **EGDMA**: ethylenegylcol dimethacrylate; **FMM**: α-methacryloyloxyethyl iminocarboxyethyloxypropylpoly(dimethylsiloxy)-butyldimethylsilane; **GPDMS**: Glycerol-functionalised polydimethylsiloxane; **HOB**: 2-hydroxybutyl methacrylate; **HPMC**: hydroxypropylmethylcellulose; **IBM**: isobomyl methacrylate; **MA**: methacrylic acid; **M3U**: αω-bis(methacryloyloxyethyl iminocarboxy ethyloxypropyl)-poly(dimethylsiloxane)-poly(trifluoropropylmethylsiloxane)-poly(methoxy-poly(ethyleneglycol)propylmethylsiloxane; **MPDMS**: Monofunctional Polydimethylsiloxane; **NMNVA**: N-methyl-N-vinyl acetamide; **NVP**: N-vinyl pyrrolidone; **PEG**: polyethylene glycol; **pHEMA**: Poly(2-hydroxyethyl methacrylate); **Si Mn**: Siloxane Macromer; **TAIC**: 1,3,5-triallyl-1,3,5-triazine-2,4,6(1H,3H,5H)-trione; **TEGDMA**: Tetraethylene Glycol Dimethacrylate; **TRIS**: propyltris(trimethylsiloxy)silane; **PVA**: poly(vinyl alcohol).data collected from manufacturers and FDA reports.

**Table 2:** Description each tear lipid layer pattern and its approximate thickness [41].

Lipid Layer Pattern	Appearance	Estimated Thickness (nm)
Meshwork	Indistinct to well defined grey, marble-like pattern. Open to tight meshwork.	≈10–50
Wave	Vertical or horizontal grey waves of good visibility. Wavy grey streak effect.	≈50–80
Amorphous	Bright blue–white appearance with no discernible features	≈80–90
Coloured fringes	Appearance of coloured interference fringes during the blink. Fringes change in colour across the surface.	>100
Abnormal coloured fringes	Discrete and small areas of highly variable coloured fringes.	Variable

**Table 3:** Pre-corneal (NIBUT) and pre-lens non-invasive break-up times (PL-NIBUT) for CL wearers (CLW) and controls.

Parameter	Group	Minimum	Maximum	Median	Mean	SD	*p*
PL-NIBUT (s)	*CLW*	2.3	31.7	5.7	7.6	6.2	**0.002**
NIBUT (s)	*Control*	2.0	45.9	8.0	10.7	9.3	

## Data Availability

The original contributions presented in the study are included in the article. Further inquiries can be directed to the corresponding author.

## Abbreviations

The following abbreviations are used in this manuscript:

CL	Contact lenses
PVA	Poly(vinyl alcohol
PVP	Poly(vinylpyrrolidone)
POTF	Pre-ocular tear film
DED	Dry eye disease
CLD	CL-induced discomfort
TFOS	The Tear Film and Ocular Surface Society
NCL	Non-contact lens
OSDI	Ocular Surface Disease Index
NIBUT	Pre-corneal non-invasive break-up time
PL-NIBUT	Pre-lens non-invasive break-up time
